# Next-generation insect pest management: genetic innovations and emerging biocontrol strategies

**DOI:** 10.3389/fpls.2026.1868879

**Published:** 2026-07-10

**Authors:** Estibaliz Sansinenea, Leandris Argentel-Martínez, Ofelda Peñuelas-Rubio, HeeJoo Hwang, Umut Toprak, Debasis Mitra, A. El-Shabasy, Sudhir Kumar Upadhyay, Jae-Ho Shin, Ugur Azizoglu

**Affiliations:** 1 Facultad de Ciencias Químicas, Benemérita Universidad Autónoma de Puebla, Puebla, Puebla, Mexico; 2 Tecnológico Nacional de México/Instituto Tecnológico del Valle del Yaqui, Bácum, Sonora, Mexico; 3 Department of Integrative Biology, Kyungpook National University, Daegu, Republic of Korea; 4 Molecular Entomology (MOLEN) Lab, Faculty of Agriculture, Department of Plant Protection, Ankara University, Ankara, Türkiye; 5 Department of Microbiology, Graphic Era (Deemed to be University), Dehradun, Uttarakhand, India; 6 Department of Biology, College of Science, Jazan University, Jazan, Saudi Arabia; 7 Research and Development Cell, Lovely Professional University, Phagwara, Punjab, India; 8 School of Chemical Engineering and Physical Sciences, Lovely Professional University, Phagwara, Punjab, India; 9 Department of Applied Biosciences, College of Agriculture and Life Sciences, Kyungpook National University, Daegu, Republic of Korea; 10 Safiye Cikrikcioglu Vocational College, Department of Crop and Animal Production, Kayseri University, Kayseri, Türkiye; 11 Genome and Stem Cell Center (GENKOK), Erciyes University, Kayseri, Türkiye

**Keywords:** crop protection, genetic pest control, smart biocontrol, transgenic insects, transgenic plants

## Abstract

Pest insects represent a major challenge to agriculture and global food security. Existing insect control methods such as chemical insecticides are under increasing scrutiny because of their environmental impacts, and once-effective methods are facing reduced acceptance due to insect pests evolving resistance and increasing regulation. This review summarizes recent advances in transgenic approaches to insect pest control over the last five years. Transgenic crops and molecular approaches play an important role in integrated pest management strategies. Integration of gene drive and transgene-generated resistance offers new strategies for targeted pest suppression, while novel platforms for delivery of dsRNA and CRISPR have broadened the range of molecular approaches. We analyze the ethical and ecological considerations, including biosafety concerns related to species interactions and gene flow. In addition, we examine the potential and limitations of RNAi and CRISPR, including regulatory challenges and public perception of genetic engineering. Synthetic biology, precision agriculture and good risk governance are central to genetic pest control strategies. Advances at the interface of biotechnology and natural systems offer a pathway toward more sustainable and resilient agricultural practices.

## 1 Introduction

Insect pests remain a major constraint in global agriculture, contributing to significant crop damage and economic losses. Recent estimates suggest that insect pests are responsible for approximately 20%–40% of global crop yield losses each year (Oerke, 2006). Traditional pest control methods, primarily based on synthetic chemical insecticides, have historically played a crucial role in reducing pest populations. However, these methods are increasingly associated with several drawbacks, including the development of resistant pest strains, toxicity to non-target organisms, environmental contamination, and risks to human health (Douglas, 2015; Tabashnik and Carrière, 2017). In response to these concerns, researchers have turned to genetic engineering as a promising and more sustainable alternative to conventional pest control. Advances in molecular biology and biotechnology have driven the development of genetically modified organisms (GMOs) encompassing both transgenic crops and insects alongside precise molecular tools such as RNA interference (RNAi) and CRISPR-Cas9 genome editing (Joga et al., 2016; Scott et al., 2018). These innovations offer highly targeted, species-specific, and potentially ecologically sustainable alternatives for integrated pest management.

One of the most widely adopted applications of genetic engineering in agriculture is the development of insect-resistant transgenic crops, particularly those expressing insecticidal proteins derived from *Bacillus thuringiensis* (*Bt*). *Bt* crops have significantly reduced the reliance on chemical insecticides for controlling major pests such as *Helicoverpa armigera*, *Plutella xylostella*, and *Spodoptera frugiperda* (Tabashnik and Carrière, 2017). Although the benefits of *Bt* crops are well documented, their widespread use has led to the emergence of resistant pest populations in some regions, highlighting the need for integrated pest resistance management strategies.

RNAi is a promising genetic technique for silencing essential genes in target insect species, resulting in developmental arrest or mortality. RNAi-based approaches offer high specificity and have shown potential for pest control with minimal impact on beneficial insects (Joga et al., 2016; Cedden and Bucher, 2025; Toprak, 2025). Although challenges remain regarding delivery systems and regulatory approval, RNAi-based biopesticides are currently under active development for field use.

More recently, CRISPR-Cas9 technology has transformed genetic engineering in insects. This system enables precise genome editing by inducing targeted double-strand breaks, allowing for gene knockouts or insertions (Champer et al., 2016). A particularly impactful application is the development of gene drive systems, which bias the inheritance of specific genes and facilitate their rapid spread in wild populations. Gene drives have been proposed for population suppression or modification of vector species such as *Anopheles gambiae*, the mosquito that transmits malaria (Hammond et al., 2016; Scott et al., 2018). However, the release of gene drives raises significant ecological and ethical concerns, including unintended effects on non-target species and gene flow. Beyond altering the pests themselves, manipulating their symbiotic microbiota offers another promising avenue for pest control. Many insects depend on symbiotic bacteria for essential functions such as nutrient synthesis and immune defense. Disrupting these microbial associations can reduce pest fitness or increase susceptibility to control measures (Douglas, 2015; Whitten et al., 2023). Although currently in its infancy, symbiont manipulation represents a novel paradigm in sustainable pest control. However, widespread implementation of these genetic technologies remains contingent upon addressing several socio-ecological and technical constraints. Regulatory frameworks vary across countries, and public perception of GMOs remains cautious. There are also legitimate scientific concerns regarding the long-term ecological impacts of releasing genetically modified insects or gene drives into open environments (Scott et al., 2018). Comprehensive risk assessments, containment protocols, and stakeholder engagement will be essential to ensure responsible innovation in this field.

Chronologically, RNAi and CRISPR represent two distinct eras in the molecular biocontrol timeline. Emerging in the late 1990s, RNAi quickly matured into a dependable framework for post-transcriptional gene silencing, grounding itself in the exploitation of endogenous eukaryotic machinery. Conversely, CRISPR-based genome editing has only recently disrupted the scene, evolving over the last decade from a prokaryotic immune defense system into a highly dynamic, programmable toolkit for direct genomic modification. While RNAi has spent decades paving the way for targeted pest management, the rapid rise of CRISPR-driven platforms signals a paradigm shift from transient transcript knockdown to permanent genomic re-engineering.

Unlike previous reviews that focus separately on RNAi or CRISPR technologies, this review provides an integrated, comparative analysis of both platforms within a single framework, emphasizing their synergistic rather than competitive roles. Key distinguishing features include: (i) a systematic side-by-side comparison of delivery platforms (HIGS, SIGS, VIGS, smRNAi) with economic feasibility assessment; (ii) detailed discussion of classical insecticide resistance mechanisms (AChE, AkP, AcP) and their intersection with RNAi/CRISPR-based strategies; (iii) explicit treatment of long-term ecological risks of gene drives, including transboundary governance challenges; and (iv) a forward-looking section integrating AI, nanotechnology, and precision agriculture into genetic pest management.

## 2 Molecular genetic approaches applied in insect pests for their control

Molecular techniques can be integrated with traditional pest management approaches as part of integrated pest management programs. For instance, RNAi sprays or genetically engineered sterile insects can be combined with cultural and biological control methods to reduce reliance on chemical pesticides and support resistance management strategies (Zotti et al., 2018). The success of these integrated strategies will depend on regulatory approval, public acceptance, and continued scientific research to ensure their efficacy and environmental safety (**Table 1**).

**Table 1:** Comparative overview of molecular genetic pest control technologies.

Technology	Mechanism	Target	Delivery	Advantages	Limitations	TRL	Field applicability	Cost	Resistance risk	Regulatory status	Environmental risks	Scalability	References
Bt crops	Cry toxins	Lepidoptera, Coleoptera	Transgenic	Proven, specific	Resistance evolution	9 (commercial)	High (global)	Medium-High	High	Approved in many countries	Low (non-target safe)	High	Tabashnik and Carrière, 2017, Gassmann and Reisig, 2023
RNAi	Gene silencing	Coleoptera, Lepidoptera	HIGS, SIGS, smRNAi	High specificity	Instability, uptake variability	6-8 (SIGS commercial)	Medium-High (sprays)	Low-Medium (sprays)	Medium	Varies by country	Low (degradable)	Medium-High	Baum et al., 2007, Rodrigues et al., 2021
CRISPR-Cas9	Genome editing	Any insect	Embryo injection	Precise, heritable	Off-target, delivery	4-6 (lab only)	Low (contained)	High	Low (engineered)	Strict/GMO regulated	High (uncertain)	Low	Taning et al., 2017, Kyrou et al., 2018
Gene Drives	Biased inheritance	Diptera	CRISPR transgenesis	Population suppression	Irreversible, spread	4-5 (cage trials)	Very Low	High	Medium	No international consensus	Very High	Very Low	Kyrou et al., 2018, Noble et al., 2019
SIT	Sterile male release	Diptera, Lepidoptera	Mass rearing, sterilization	Species-specific, proven	Fitness cost	9 (classical)	High (area-wide)	Medium	Very Low	Approved for SIT	Low	Medium	Dyck et al., 2021, Kandul et al., 2022
Symbiont Engineering	Effector expression	Hemiptera, Diptera	Symbiont delivery	Reversible, contained	Instability, regulation	4-5 (experimental)	Very Low	Medium-High	Medium	Unclear	Low-Medium	Low	Whitten et al., 2016, Leonard et al., 2020

### 2.1 Genetically engineered insects

Modern genetic engineering approaches in insects encompass both transgenic technologies and genome-editing tools such as CRISPR-Cas9. While CRISPR is a key enabler, its broader applications are detailed in Section 2.4. Recent advances in genetic engineering have enabled the development of novel strategies to control insect pests and disease vectors that threaten agriculture and public health. Among these, gene drives and transgenic resistance to pathogens have shown significant promise in both laboratory and limited field applications.

Gene drives are genetic constructs that bias inheritance patterns, allowing specific genes to spread rapidly through wild populations, thereby overriding traditional Mendelian inheritance. A notable example is the CRISPR-Cas9-based homing gene drive developed by Kyrou et al. (2018), which targets the female-specific exon of the doublesex gene in *Anopheles gambiae*, a key malaria vector in Africa. This modification induced complete female sterility in drive homozygotes without affecting males, leading to the collapse of mosquito populations in caged experiments within 7–11 generations and demonstrating the potential of gene drives for malaria vector control. Subsequent work has focused on improving the robustness of gene drives by addressing resistance allele formation through strategies such as multiplexed guide RNAs and optimized cleavage site design, as comprehensively reviewed by Champer et al. (2020). In addition to population suppression, genetic engineering has been employed to render mosquitoes refractory to pathogens, thereby reducing disease transmission without necessarily decreasing vector populations. Isaacs et al. (2012) developed transgenic *Anopheles stephensi* expressing single-chain antibodies against *Plasmodium falciparum*, which effectively inhibited parasite development in the mosquito midgut. Similarly, Dong et al. (2018) introduced synthetic antimicrobial peptides into transgenic mosquitoes to disrupt Plasmodium development, resulting in significant parasite inhibition. Carballar-Lejarazú and James (2017) reviewed transgenic approaches based on the expression of multiple anti-parasitic effectors in *A. gambiae*, highlighting their potential to reduce *Plasmodium* infection. More recently, Carballar-Lejarazú et al. (2023) experimentally combined anti-Plasmodium effector genes with a CRISPR-based homing gene drive in *A. gambiae*, demonstrating efficient population modification and robust resistance to Plasmodium infection.

Although these genetic control technologies show considerable promise, their implementation raises concerns regarding ecological safety, public acceptance, and regulatory oversight. In response, the World Health Organization (WHO, 2021) has issued guidance for the phased testing of genetically modified mosquitoes, emphasizing the importance of thorough risk assessments, community engagement, and ethical transparency.

### 2.2 Transgenic crops and pest resistance

Genetic engineering has been widely applied in agriculture to create transgenic crops with traits superior to those of wild-type varieties. This involves the integration of specific DNA sequences, known as transgenes, into the germplasm of crop plants to confer desirable characteristics (Kumar et al., 2020). Once incorporated, these transgenes can be expressed throughout the plant (Iqbal et al., 2021), eliminating the limitations of external application and offering numerous benefits such as improved shelf life, higher yields, and enhanced resistance to pests, extreme temperatures, and drought (Zhao et al., 2021). This process, known as transgenesis, is typically achieved through methods such as the biolistic (gene gun) technique or *Agrobacterium tumefaciens*-mediated transformation (Sood and Lal, 2023). However, the introduction of foreign genes also raises concerns, including the potential for gene flow to wild relatives and the horizontal transfer of antibiotic resistance markers. To address these risks, alternative strategies such as cis-genesis and intra-genesis have been developed. Cis-genesis involves inserting an identical copy of a gene from the same species, while intra-genesis involves combining genetic elements from different genes within the same species (Kumar et al., 2020).

Genome editing has further advanced crop biotechnology by enabling highly precise modifications at specific genomic loci. Tools such as zinc finger nucleases (ZFNs), transcription activator-like effector nucleases (TALENs), and the CRISPR/Cas system allow targeted mutation or gene insertion with minimal off-target effects (Kumar et al., 2020; Abdul Aziz et al., 2022). To date, most genome-edited and transgenic crops approved for commercialization have focused on agronomic traits, such as nutritional improvement and stress tolerance, rather than direct engineering of insect resistance (Abdul Aziz et al., 2022).

Among the primary applications of transgenic crops, including resistance to herbicides, diseases, and abiotic stress, insect resistance remains one of the most significant. To date, nine insect-resistant transgenic crops have been approved for commercial cultivation. Maize is the most widely cultivated, but other approved crops include cotton, potato, soybean, rice, sugarcane, poplar, brinjal (eggplant), and tomato (Kumar et al., 2020). The most well-known insecticidal genes are the *cry* genes derived from *Bt*, which produce proteins that act as bioinsecticides. The development and widespread adoption of *Bt* crops represent a crucial step in pest management (Ortiz and Sansinenea, 2023). These crops have significantly reduced dependence on chemical insecticides, resulting in substantial economic benefits and a lower environmental footprint (Brookes, 2022).

**Figure 1:**
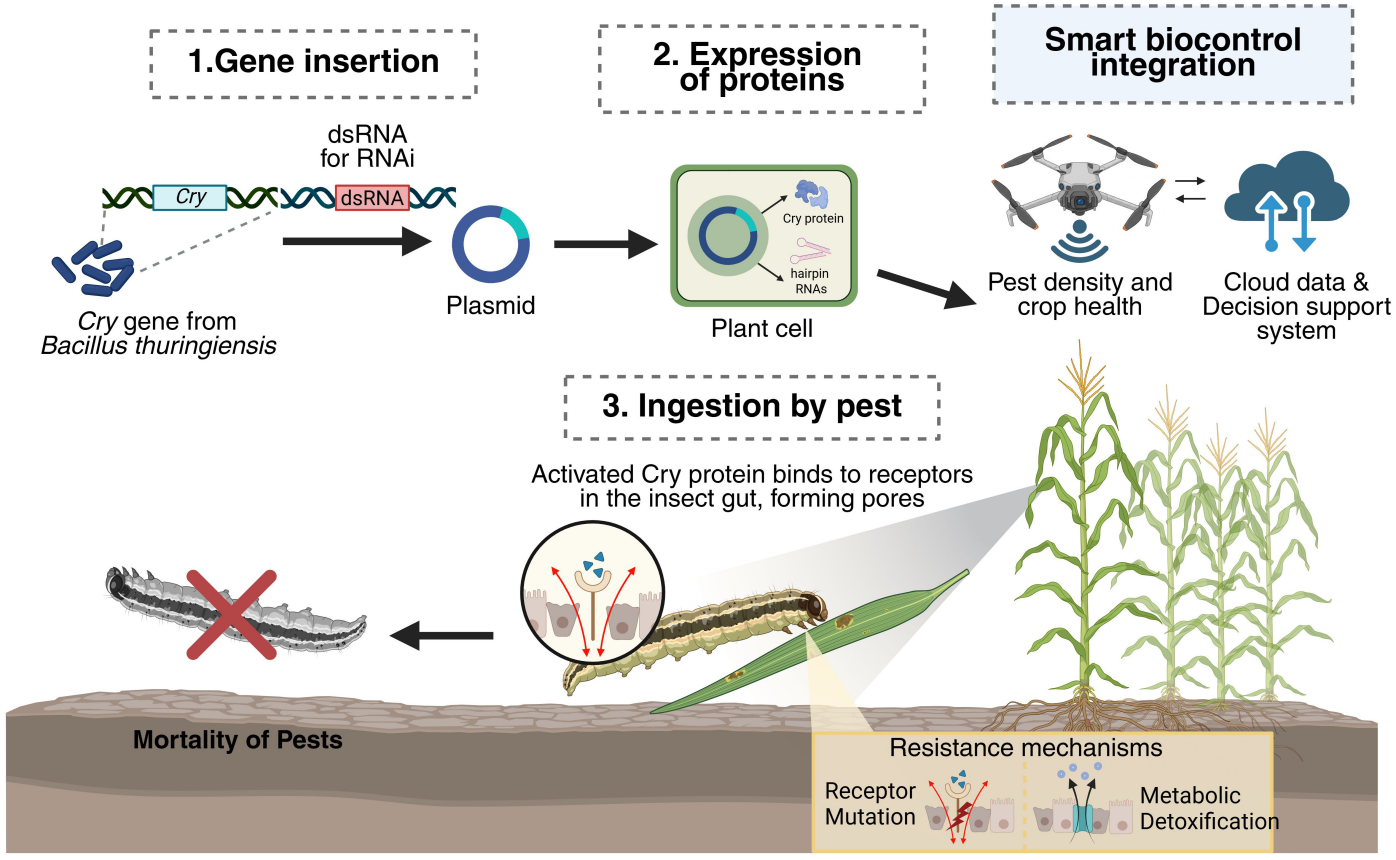
Mode of action of *Bt.* In addition to plant-encoded dsRNA from transgenes (HIGS), exogenous dsRNA can be introduced directly via spray-induced gene silencing (SIGS), as indicated by the dashed arrow in the figure.

Cry proteins produced by *Bt* crops bind to specific receptors in the gut of susceptible insects, leading to pore formation, gut paralysis, and ultimately insect death (**Figure 1**) (Pardo-López et al., 2013). As illustrated in **Figure 1**, the mode of action begins with the ingestion of Cry protoxins, which are solubilized in the alkaline midgut environment and proteolytically cleaved into active toxins. These activated toxins bind specifically to cadherin and aminopeptidase-N receptors on the brush border membrane, promoting oligomerization and subsequent binding to ABC transporters. This interaction triggers insertion of the toxin into the membrane, forming lytic pores that disrupt ion homeostasis, cause gut paralysis, and ultimately lead to bacterial septicemia and insect death. This specificity allows *Bt* crops to target harmful pest species without affecting beneficial insects or mammals. Several companies, including Monsanto and Mycogen, developed novel Bt-based insecticidal products by modifying promoters, antibiotic resistance markers, transformation and tissue culture systems, and by engineering new insecticidal proteins (Castagnola and Jurat-Fuentes, 2012). One notable example is the New Leaf potato variety, which expressed the cry3A toxin gene. Developed in 1995 by NatureMark, a Monsanto affiliate, it was the first commercially available *Bt* crop (Castagnola and Jurat-Fuentes, 2012). Subsequently, other *Bt* crops were commercialized, including maize, cotton, tomato, tobacco, rice, and broccoli. Despite widespread adoption, several major pests including the American bollworm (*Helicoverpa zea*) and fall armyworm (*Spodoptera frugiperda*) continue to cause significant economic damage as they have evolved resistance to certain Cry toxins, feeding successfully on *Bt* crops in many regions (Tabashnik and Carrière, 2017; Gassmann and Reisig, 2023). This persistent threat underscores the urgent need for complementary technologies such as RNAi and CRISPR-Cas9 to sustain effective pest management.

However, the widespread adoption of these transgenic crops has generated considerable debate regarding their safety for human health (Kedisso et al., 2023; Gassmann and Reisig, 2023).

Beyond crops producing *Bt* toxins, there are emerging transgenic technologies that develop additional methods for insect control and serve to slow the evolution of resistance against these products. These technologies use transgenic expression of plant-derived or heterologous protease inhibitors and lectins that affect the digestion, nutrient absorption, and physiology of the insect’s gut. Although these systems have yet to gain the commercial appeal of *Bt*-based systems, they offer important proof-of-concept for alternative transgenic insect defense strategies. Likewise, plant-mediated RNA interference (HIGS) represents a new, promising option for pest control without the direct use of *Bt* toxins. HIGS technology involves creating transgenic crops that produce double-stranded RNA that will target essential insect genes. HIGS represents a species-specific approach to pest management, and it can be incorporated with other insecticidal traits. The use of CRISPR technology to create genome-edited crops also opens up new possibilities for improving pest resistance by allowing researchers to modify plant susceptibility genes and/or plant defense pathways. Depending on the regulatory framework, these crops may be considered “non-transgenic-like” crops and could facilitate adoption. This term refers to genome-edited plants that contain small insertions or deletions (indels) or single nucleotide substitutions resulting from non-homologous end joining (NHEJ) repair, without the integration of foreign DNA (transgenes). In regulatory frameworks such as those of Argentina, Japan, and the United States, such crops may be exempt from GMO regulations if they do not contain foreign genetic material (Kumar et al., 2020; Abdul Aziz et al., 2022). In contrast, the European Union classifies all genome-edited organisms as GMOs regardless of transgene presence, requiring full risk assessment and traceability (Kedisso et al., 2023).

*Bt* maize and cotton have been particularly successful and are now widely cultivated across North and South America, Asia, and Africa. According to the ISAAA, over 120 million hectares of biotech crops were planted globally in 2022, with insect resistance being the most dominant trait (ISAAA, 2023). Studies have shown that these crops not only improve yields but also reduce the need for chemical pesticides, supporting more sustainable agricultural practices (Ortiz et al., 2025).

The resistance development in some pest species, such as *H. armigera* and *S. frugiperda*, poses considerable difficulties for extending the long-term effectiveness of *Bt* crops. The mechanisms through which these lepidopteran pests develop resistance that have been identified include mutations and mis-splicing of the genes for the receptors of Cry toxins, specifically the genes that encode ATP binding cassette (ABC) transporters and cadherin proteins located in the midgut. Mutations in these genes decrease Cry binding and provide practical resistance when the pests are exposed to environmental conditions in the field (Jurat-Fuentes et al., 2011; Xie et al., 2026). In *H. armigera*, multiple mutant alleles of cadherin have been reported to confer Cry1A resistance, while disruptions in ABCC2 and ABCA2 transporters were reported as being associated with Cry1Ac and Cry2Ab resistance (Niu et al., 2024; Xie et al., 2026). Similarly, *S. frugiperda* populations are developing resistance to Cry1F through mutations in their receptors for Cry1F, in addition, they exhibit cross-resistance among the Cry proteins, making resistance management increasingly difficult for pyramided *Bt* maize hybrids. Resistance is also conferred through metabolic pathways that aid in detoxifying Cry toxins, such as increased cytochrome P450 monooxygenases and other enzymes, which help increase survival chances when subjected to selection pressure. To address these adaptive responses, pest resistance management is being pursued through a combination of integrated strategies, including deployment of high-dose/refuge, pyramiding of Cry toxins, and incorporation of complementary technologies such as RNA interference (RNAi). Use of pyramided *Bt* genes and RNAi traits has been shown to delay the evolution of resistance due to the fact that they act independently against resistance alleles, while structured refuges of non-*Bt* host plants maintain populations of susceptible insects and decrease selection pressure (Tabashnik and Carrière, 2017). Therefore, the combination of these diverse strategies is the key to maintaining the effectiveness of *Bt* crops against rapidly evolving pest populations. RNAi technology has emerged as a complementary approach to *Bt* technology, offering highly specific gene silencing in target pests. For instance, transgenic maize producing double-stranded RNA (dsRNA) targeting corn rootworm (*D. v. virgifera*) results in mRNA suppression and reduced feeding damage (Baum et al., 2007). This gene-silencing technique involves engineering plants to express dsRNA molecules that form hairpin structures (hpRNA), leading to RNAi-based inhibition in the pest (Shukla et al., 2016). For example, Monsanto’s MON87411 maize expresses dsRNA targeting western corn rootworm. Similarly, tobacco has been genetically modified using RNAi to confer resistance against the green peach aphid (*Myzus persicae*) (Mao and Zeng, 2023; Christiaens et al., 2022) (**Table 2**).

**Table 2:** Field applications and performance metrics of genetic pest control approaches.

Application	Target organism	Control strategy	Deployment region	Observed outcome	References
*Bt* maize (*MON87411*)	*D. virgifera virgifera* (corn rootworm)	Bt Cry3Bb1 + RNAi (Snf7 gene silencing)	USA, Argentina	>95% reduction in feeding damage; delayed resistance development	Bachman et al., 2013
Gene drive in *Anopheles gambiae*	*A. gambiae* (malaria vector)	CRISPR-based gene drive targeting *doublesex* gene	Lab (UK), cage experiments	Population collapse within 7–11 generations	Kyrou et al., 2018
RNAi foliar spray on potato	*Leptinotarsa decemlineata* (Colorado beetle)	dsRNA targeting *Actin* and *V-ATPase*	Field trials in North America	>80% mortality; reduced reproductive fitness	Zotti et al., 2018
SIT in *Ceratitis capitata*	Mediterranean fruit fly	Radiation-sterilized males	Mediterranean countries	Area-wide pest suppression; reduced insecticide use	Dyck et al., 2021
Transgenic *Anopheles stephensi*	*Plasmodium* transmission in mosquitoes	Expression of anti-*Plasmodium* antibodies	India (contained facility)	85%–95% inhibition of parasite development in mosquito gut	Isaacs et al., 2012
*Wolbachia*-infected *Aedes aegypti*	Dengue virus vector	Cytoplasmic incompatibility	Australia, Brazil, Indonesia	Dengue incidence reduced by up to 77%; stable *Wolbachia* transmission in mosquito populations	WHO, 2021

Comprehensive risk assessments conducted by organizations such as the European Food Safety Authority (EFSA) have confirmed that *Bt* crops are safe for non-target organisms and human health. Nevertheless, continued ecological monitoring, studies on the development of resistance and its management are essential to detect any unforeseen effects and maintain public trust in biotechnology (EFSA, 2018; Niu et al., 2024; Xie et al., 2026).

### 2.3 RNAi based pest control

RNA interference (RNAi) is a conserved eukaryotic mechanism of post-transcriptional gene regulation, involving sequence-specific mRNA degradation triggered by dsRNA. Following its initial discovery in *Caenorhabditis elegans* (Fire et al., 1998), RNAi has evolved from a functional genomics tool into a translational technology with direct relevance for species-specific pest management. The first demonstration in insects was in Drosophila melanogaster, where dsRNA targeting the frizzled gene caused developmental defects (Kennerdell and Carthew, 1998) and was later extended to other genes with improved delivery methods (Kennerdell and Carthew, 2000). These pioneering studies paved the way for applications across diverse insect taxa (Belles, 2010) and the development of RNAi-based biopesticides (Cedden and Bucher, 2025; Toprak, 2025).

**Figure 2:**
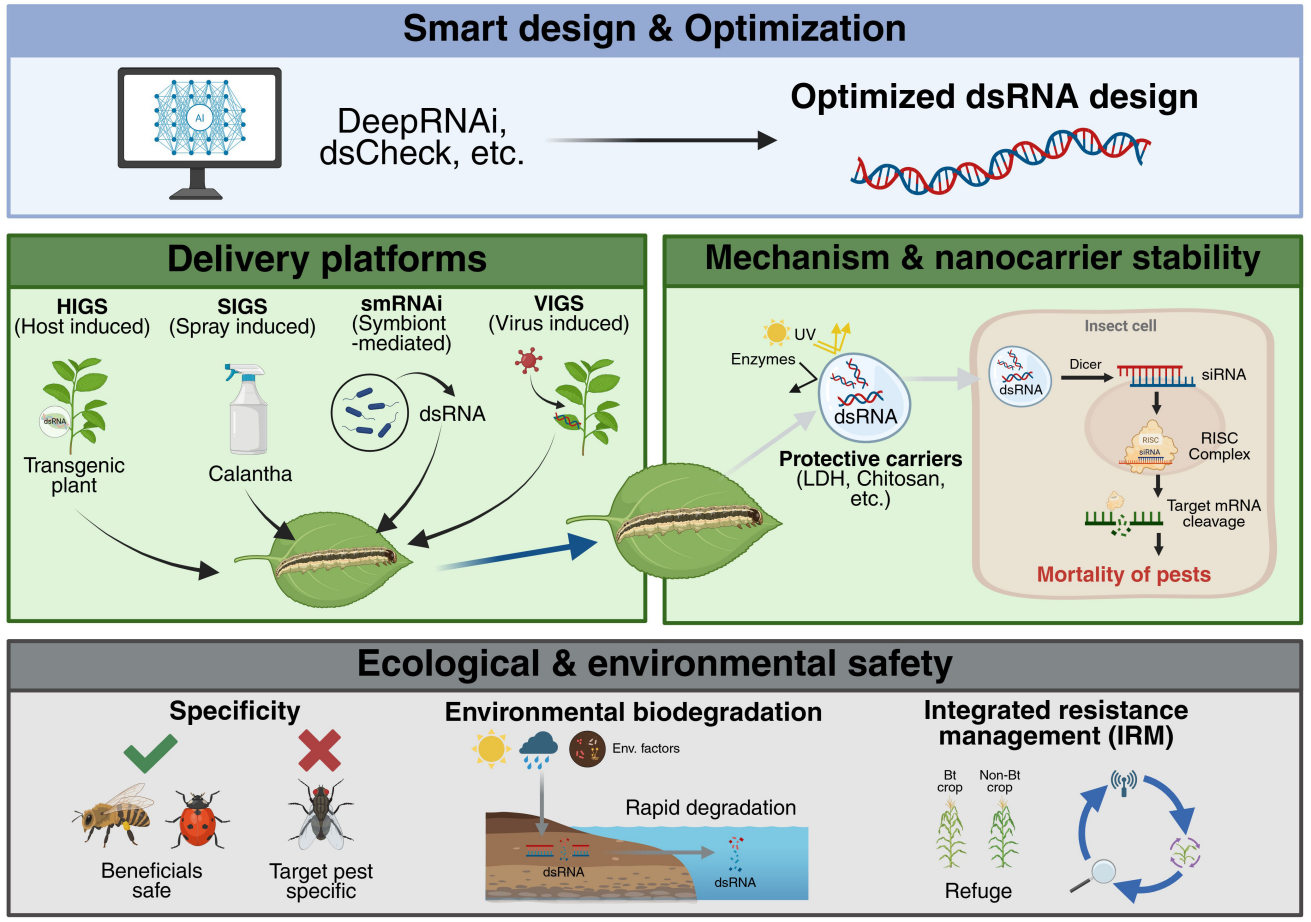
RNAi-based pest insect control. Protective nanocarriers include LDH, chitosan, lipid nanoparticles, and liposomes.

The holistic pipeline of translational RNAi-based pest management encompasses computational design, diverse delivery modalities, cellular processing, and environmental safety frameworks, as comprehensively mapped in **Figure 2**. As illustrated in the ‘Smart design & Optimization’ panel, the pipeline initiates with in silico screening using advanced bioinformatics suites and artificial intelligence predictors, such as DeepRNAi and dsCheck, to generate optimized dsRNA sequences that maximize target silencing while minimizing off-target alignment. These sequence-verified molecules are subsequently deployed across distinct ‘Delivery platforms, ’ which include host-induced gene silencing (HIGS) via transgenic plants, spray-induced gene silencing (SIGS) using commercial formulations like Calantha™, symbiont-mediated delivery (smRNAi), and transient virus-induced vectors (VIGS). To overcome the significant environmental liabilities of naked dsRNA specifically ambient UV degradation and extracellular enzymatic cleavage protective nanocarriers such as Layered Double Hydroxides (LDH) and chitosan, lipid nanoparticles, and liposomes, are leveraged to reinforce ‘Mechanism & nanocarrier stability’. These nanocarriers enhance dsRNA persistence, cellular uptake, and protection against enzymatic degradation. Each step depicted in **Figure 2** from in silico target selection and dsRNA optimization to delivery platforms, cellular processing, and environmental safety assessment represents a critical control point that determines the success or failure of RNAi-based pest management in the field. Upon successful cellular internalization into the insect host, the exogenous dsRNA enter the intracellular RNAi machinery. Here, long dsRNA is recognized and enzymatically cleaved by Dicer-2 into discrete small interfering RNAs (siRNAs). Following the thermodynamic unwinding of the siRNA duplex, the passenger strand is discarded, allowing the guide strand to precisely program the Argonaute-containing RNA-induced silencing complex (RISC) to bind and cleave complementary target mRNA, effectively shutting down translation and driving pest mortality. Finally, as evaluated in the ‘Ecological & environmental safety’ matrix, this molecular cascade ensures strict target specificity to spare key beneficial insects (e.g., honey bees and ladybugs), exhibits rapid environmental biodegradation to avoid soil or aquatic persistence, and requires rigorous integration with resistance management (IRM) strategies, such as structured non-*Bt* refuges, to sustain field durability (Hutvágner and Zamore, 2002; Martinez et al., 2002). Unlike the emerging CRISPR-based RNA targeting approaches (crRNAi), which utilize programmable Cas13 or Cas12a nucleases to cleave transcript targets directly without relying on endogenous host machinery, classical eukaryotic RNAi is fundamentally constrained by the host’s cellular physiology. The systemic nature of RNAi varies drastically across insect orders; while coleopterans exhibit robust systemic RNAi mediated by explicit endosomal uptake pathways and SID-1-like transmembrane channel proteins, lepidopterans often display marked refractoriness. This biological barrier stems from high titers of extracellular dsRNases in the lepidopteran gut fluid, alkaline pH profiles that promote dsRNA degradation, and deficient endosomal escape, collectively undermining the reproducibility and field-level efficacy of unformulated dsRNA triggers.

RNAi delivery in insects has evolved from laboratory microinjection-based methods to scalable, field-oriented platforms, encompassing plant-mediated expression systems (Host-induced gene silencing: HIGS), sprayable dsRNA formulations (Spray-induced gene silencing: SIGS), symbiont-mediated RNAi (smRNAi), and virus-induced gene silencing (VIGS). Each method offers distinct advantages and limitations based on pest biology, tissue tropism, and ecological factors.

To systematically evaluate these delivery platforms, HIGS, SIGS, and VIGS must be contrasted across efficiency, cost-structures, and regulatory hurdles. HIGS provides continuous, tissue-specific protection across generations, eliminating the labor and material costs of repeated applications; however, its economic feasibility is bottlenecked by the immense upfront capital required for transgenic trait development and navigating complex regional GMO deregulaton. Conversely, SIGS offers an agile, non-transgenic alternative with faster market entry, but faces substantial economic constraints due to the high operational costs of scaling high-purity dsRNA synthesis and the requirement of sophisticated nanocarriers (e.g., layered double hydroxides or chitosan) to prevent premature environmental degradation. VIGS remains predominantly restricted to transient functional genomics and screening pipelines. While highly efficient in yielding rapid phenotypes without stable transformation, the inherent biosafety risks of horizontal viral transmission and potential persistence in non-target agroecological webs significantly limit its direct commercial adoption for field-scale pest suppression.

*Transgenic plants (Host-induced gene silencing, HIGS)*: Transgenic plants (Host-induced gene silencing, HIGS): Genetically engineered plants express long or hairpin dsRNAs targeting essential pest genes. First validated by Baum et al. (2007) in maize against *D. v. virgifera* V-ATPase subunits, this approach achieved strong gene knockdown and larval mortality comparable to Bt lines. Subsequent studies have demonstrated the efficacy of HIGS across multiple crop-pest systems, including MON87411 maize targeting the *Snf7* gene (Bachman et al., 2013; Narva et al., 2025). Similarly, plastid-transformed potato plants expressing dsRNA targeting β-actin conferred complete protection against the Colorado potato beetle *Leptinotarsa decemlineata* through robust in plant RNAi (Zhang et al., 2015).

Beyond coleopterans, transgenic strategies for lepidopteran pests (*H. armigera*, *Spodoptera* spp., *Mamestra configurata*) show reduced efficacy due to high gut nuclease activity, poor dsRNA uptake, and weak systemic spread (Toprak et al., 2014). Arabidopsis thaliana expressing dsRNA for CYP6AE14 impaired *H. armigera* detoxification of the plant defense compound gossypol, resulting in reduced larval growth and weight despite incomplete mortality, thereby enhancing the effectiveness of existing plant defenses (Mao and Zeng 2023). Plant-derived long dsRNAs, not siRNAs, are the active triggers in insects, with efficacy limited by the absence of RNA-dependent RNA polymerase (RdRP) and restricted systemic RNAi (Gordon and Waterhouse, 2007; Niu et al., 2024). While HIGS offers continuous, species-specific control, adoption is limited by regulatory complexity and public resistance to GM crops.

*Spray-induced gene silencing (SIGS)*: This relies on the application of exogenous dsRNA to plant surfaces, which is subsequently ingested by herbivorous insects during feeding. A key limitation of this approach is the environmental instability of dsRNA under field conditions, an issue that has driven the development of protective carrier systems, as discussed in detail below.

In potato, foliar dsRNA sprays targeting Actin and V-ATPase induced high mortality in *L. decemlineata* (Zotti et al., 2018). Calantha™ represents a benchmark spray-induced RNAi product, in which dsRNA targeting the proteasome subunit PSMB5 achieves consistent field performance while maintaining a favorable non-target safety profile (Rodrigues et al., 2021; Narva et al., 2025). Similarly, a chitosan-formulated dsRNA targeting Snf7 in *Frankliniella occidentalis* provided >80% control (Khan et al., 2025).

New candidates include Teranura™ (targeting the two-spotted spider mite *Tetranychus urticae*) and Vadescana™ (designed for the parasitic mite *Varroa destructor*), both showing improved stability and uptake (McGruddy et al., 2024; Bortolin et al., 2025). In V. destructor, silencing calmodulin reduced reproduction while sparing honey bee brood (McGruddy et al., 2024; Bortolin et al., 2025). Recent transcriptomics has identified salivary gland targets for RNAi (Becchimanzi et al., 2025). SIGS has also been extended to phytophagous mites, with dsRNA against chitin synthase 1 (CHS1) reducing *Amphitetranychus viennensis* populations and leaf damage under semi-field conditions (Duan et al., 2025). These advances highlight formulation chemistry and delivery design as key determinants of field success.

*Symbiont-mediated RNAi (smRNAi):* Engineered gut symbionts capable of producing dsRNA represent a biologically contained and continuous delivery system within the insect host. Pioneering efforts have focused on *Rhodococcus rhodnii* in *Rhodnius prolixus* and *Sodalis glossinidius* in tsetse flies, where symbionts were genetically modified to express dsRNA targeting essential insect genes (Whitten et al., 2016). Whitten and Dyson (2017) expanded the concept, addressing host–symbiont compatibility, transmission, tissue tropism, and biosafety, and proposed smRNAi as a versatile tool, especially in Hemiptera and Diptera.

Recent work has extended smRNAi to *F. occidentalis* (Thysanoptera), *A. mellifera* (Hymenoptera), and *A. stephensi* (Diptera) using species-specific symbionts (BFo2, *Snodgrassella alvi*, *Serratia fonticola*) (Figueiredo et al., 2025). These can be engineered with optimized promoter systems and integrated expression cassettes, particularly in rnc-deficient strains to prevent dsRNA degradation. smRNAi protects dsRNA from gut nucleases, enables prolonged *in vivo* expression, and in social insects supports colony-wide spread via trophallaxis or frass.

Beyond pest gene silencing, smRNAi has suppressed bee pathogens (*V. destructor*, *Nosema ceranae*, and deformed wing virus) without colonizing non-targets (Leonard et al., 2020; Whitten et al., 2023). Deployment remains limited by regulatory concerns over containment and horizontal gene transfer, prompting development of chromosomal integration and kill-switch biosafety systems (Figueiredo et al., 2025).

*Virus-Induced Gene Silencing (VIGS):* Recombinant viral vectors such as Tobacco Rattle Virus (TRV) enable efficient, transient knockdown of plant genes without stable transformation. TRV-based systems have been applied to herbaceous and woody plants (*Styrax japonicus*, Glycine max), achieving 65–95% silencing depending on delivery method and *Agrobacterium* density (Deng et al., 2025). VIGS is also being adapted for epigenetic applications, where virus-delivered small RNAs trigger RNA-directed DNA methylation (RdDM), enabling heritable silencing without transgenesis (Zulfiqar et al., 2023). In insects, VIGS-like approaches using recombinant Flock House Virus (FHV) in D. melanogaster have demonstrated systemic gene silencing in species refractory to dsRNA uptake (Taning et al., 2018), though technical and biosafety constraints currently limit broader application. In the context of pest management, VIGS is primarily used as a functional genomics tool to identify and validate plant or insect targets prior to the development of RNAi-based control strategies.

To systematically evaluate these delivery platforms, HIGS, SIGS, and VIGS must be contrasted across efficiency, cost structures, and regulatory hurdles. HIGS provides continuous, tissue-specific protection across generations, eliminating the labor and material costs of repeated applications; however, its economic feasibility is bottlenecked by the immense upfront capital required for transgenic trait development and navigating complex regional GMO deregulation. Conversely, SIGS offers an agile, non-transgenic alternative with faster market entry, but faces substantial economic constraints due to the high operational costs of scaling high-purity dsRNA synthesis and the requirement of sophisticated nanocarriers (e.g., layered double hydroxides or chitosan) to prevent premature environmental degradation. VIGS remains predominantly restricted to transient functional genomics and screening pipelines. While highly efficient in yielding rapid phenotypes without stable transformation, the inherent biosafety risks of horizontal viral transmission and potential persistence in non-target agroecological webs significantly limit its direct commercial adoption for field-scale pest suppression.

*Determinants of RNAi Efficacy in Insects:* RNAi outcomes are shaped by physiological, biochemical, environmental, and regulatory factors. Despite conservation of the core RNAi machinery, functional outcomes differ substantially among insect orders, with robust and systemic responses in many coleopterans, inconsistent efficacy in hemipterans, and frequent refractoriness in lepidopterans (Terenius et al., 2011; Huvenne and Smagghe, 2010; Toprak et al., 2013; Koo et al., 2023). Off-target effects, although reduced by rigorous sequence design, remain a concern, particularly when short siRNAs share partial homology with non-target transcripts. Furthermore, extracellular dsRNases in lepidopteran gut fluid, alkaline pH profiles that promote dsRNA degradation, and inefficient endosomal escape collectively undermine experimental reproducibility, necessitating the use of protective nanocarriers (e.g., chitosan, layered double hydroxides) for field deployment (Mitter et al., 2017; Darrington et al., 2025).

A major determinant is dsRNA uptake and systemic spread, which varies greatly across taxa. Coleopterans such as *T. castaneum* and *L. decemlineata* show robust systemic and parental RNAi, aided by SID-like transporters and clathrin-mediated endocytosis (Huvenne and Smagghe, 2010; Doğan et al., 2021; Güney et al., 2021). In *T. castaneum*, Bucher et al. (2002) first demonstrated near-complete zygotic gene knockdown in offspring after maternal dsRNA injection. Lepidopterans, lacking SID homologs, respond poorly (Terenius et al., 2011; Toprak et al., 2013). Gut pH, peritrophic matrix permeability, and nuclease activity further limit efficacy; silencing dsRNase2/3 can improve responses in Hemiptera and Thysanoptera (Zotti et al., 2018; Khan et al., 2025).

Selecting essential genes with low redundancy maximizes impact. Bioinformatics tools such as dsCheck, siDirect, and dsRNA engineer, and AI-based predictors like DeepRNAi, optimize target choice while minimizing off-targets (Chen et al., 2023; Cedden and Bucher, 2025). Expanding beyond mortality to developmental, reproductive, diapause, or stress-response genes can yield sustainable control. Classical insecticide resistance often arises from point mutations in acetylcholinesterase (AChE), which confers resistance to organophosphates and carbamates, or from elevated activity of metabolic enzymes such as cytochrome P450 monooxygenases, esterases, and glutathione-S-transferases (GSTs). In the context of Bt resistance, reduced activity of midgut alkaline phosphatases (AkP) and acid phosphatases (AcP) has been implicated in diminished Cry toxin binding and pore formation (Jurat-Fuentes et al., 2011; Xie et al., 2026). RNAi offers a complementary strategy to suppress resistance-associated genes; for example, silencing P450 monooxygenases or esterases in resistant insect strains has been shown to resensitize populations to chemical insecticides. Conversely, CRISPR has been used to functionally validate resistance alleles such as mutations in cadherin, ABCC2, and ABCA2 transporters in H. armigera and S. frugiperda, providing mechanistic confirmation that can guide the selection of RNAi targets for resistance management (Niu et al., 2024). In addition, silencing diapause associated genes in *L. decemlineata* disrupted lipid metabolism and overwintering success (Cedden et al., 2024).

UV light, rainfall, and soil microbes degrade dsRNA, while temperature extremes reduce efficacy (Darrington et al., 2025). Protective nano-carrier technologies, including layered double hydroxide (LDH) nanosheets, chitosan nanoparticles, and carbon quantum dots, enhance dsRNA persistence, stability and uptake under field conditions (Mitter et al., 2017; Das et al., 2015; Yang et al., 2022).

RNAi products require ERA frameworks addressing environmental fate, non-target effects, and horizontal gene transfer. EFSA and OECD now provide guidance on degradation kinetics, non-target arthropod exposure, and tiered lab–field testing. Romeis and Widmer (2020) emphasize harmonized protocols for non-target risk assessment, persistence studies, and cross-jurisdictional data requirements.

Large-scale dsRNA production via *E. coli* HT115*, Corynebacterium glutamicum*, or *Yarrowia lipolytica* offers cost advantages over *in vitro* transcription. Process optimization temperature control, induction timing, streamlined purification can cut costs by >50% without yield loss (Papić et al., 2018).

RNAi can complement biological and chemical controls. For instance, foliar dsRNA targeting *L. decemlineata* digestive enzymes, combined with the predatory stink bug *Podisus maculiventris*, enhanced mortality beyond single treatments (Taning et al., 2017). Such integration supports pesticide reduction and preserves beneficial insects and other non-target organisms.

Resistance evolution remains a concern. *L. decemlineata* selected for dsRNA resistance retained susceptibility to multiple insecticide classes (Mishra et al., 2021), supporting rotation with conventional chemistries. Resistance selection pressures differ fundamentally between delivery modes, with externally applied dsRNA creating spatially and temporally heterogeneous exposure, whereas constitutive in plant expression can generate sustained selection across pest generations. Delivery-specific IRM should include refuges, mode-of-action rotation, and modeling. Both innate refractoriness (uptake barriers, nucleases) and adaptive resistance (target-site mutations, pathway modulation) require integrated monitoring and mitigation (Narva et al., 2025).

### 2.4 CRISPR-Cas9 and genome editing in insects

In contrast to RNAi, which has advanced into field-deployable platforms for insect pest control, CRISPR-Cas9 should not be regarded as a direct biocontrol method. CRISPR-Cas9 induces targeted double-strand breaks (DSBs) that are primarily repaired by error-prone non-homologous end joining (NHEJ), leading to frameshift mutations and gene knockouts. In contrast, homology-directed repair (HDR) enables precise gene insertion or allele replacement but remains inefficient in most insect systems due to the low abundance of donor templates and limited activity of HDR machinery in non-dividing cells (Taning et al., 2017). Off-target effects, driven by mismatched tolerance of the guide RNA, can be mitigated using paired guide RNAs, high-fidelity Cas9 variants (e.g., SpCas9-HF1, eSpCas9), truncated single-guide RNAs, or base editing strategies that minimize DSB formation altogether.

Beyond Cas9, other CRISPR systems have expanded the genome editing toolbox for insect applications. Cas12a (Cpf1) recognizes T-rich protospacer adjacent motifs (PAMs) and generates staggered cuts, facilitating multiplexed gene editing. CasΦ, a compact Cas enzyme from bacteriophages, offers efficient editing in size-constrained viral vectors. Base editors (cytosine and adenine base editors) enable single-nucleotide substitutions without DSBs, while prime editors mediate targeted insertions, deletions, and all possible base conversions without requiring donor DNA templates (El-Awaad and Merzendorfer, 2025).

Delivery of CRISPR components into insect embryos or somatic tissues remains a major technical barrier. Common delivery methods include microinjection of plasmid DNA, mRNA, or ribonucleoprotein (RNP) complexes; viral vectors (e.g., adeno-associated virus, baculovirus); lipid nanoparticles; and cell-penetrating peptides. RNP delivery is preferred for minimizing off-target effects and avoiding prolonged expression of Cas9, as the complex degrades rapidly after achieving genome editing. Rather, it represents an enabling technology that underpins next-generation pest management strategies by accelerating functional genomics, validating molecular targets, and facilitating the development of genetic innovations such as gene drives, sterile insect techniques (SIT), and genetically modified resistant crops (Taning et al., 2017; Kyrou et al., 2018; Faizal et al., 2024). In this sense, CRISPR functions as a molecular engine that expands the toolbox for designing smart and sustainable insect control interventions, even though most applications remain restricted to laboratory or contained environments.

CRISPR is also being used to strengthen plant defenses against herbivorous insects. By knocking out susceptibility genes or altering metabolic pathways, host plants can be made less suitable for pests (Moon et al., 2022). For example, editing serotonin biosynthesis pathways in rice reduced plant hopper feeding and growth (Faizal et al., 2024). Such host-side interventions represent a complementary avenue for crop protection within integrated pest management.

Beyond Cas9, novel systems such as Cas12, CasΦ, base editors, and prime editors are being developed to achieve greater precision and versatility. These next-generation platforms may overcome current barriers in efficiency and specificity, broadening their applicability in insects. Innovative delivery strategies, including lipid nanoparticles and cell-penetrating peptides, are also being explored to reduce technical limitations in insect embryo manipulation (El-Awaad and Merzendorfer, 2025).

Despite exciting laboratory successes, CRISPR-based interventions face significant biosafety and ecological challenges. Off-target effects, self-propagating gene drives, and potential irreversible impacts on non-target populations remain major concerns (Taning et al., 2017; Romeis and Widmer, 2020). Strategies such as self-limiting or reversible drives and molecular kill-switches are being developed to mitigate these risks (Oberhofer et al., 2020).

Regulatory frameworks differ across regions, with the United States, European Union, and Australasia adopting distinct approaches to genome-edited insects (Zahoor et al., 2024). Harmonized international policies and transparent risk assessments will be essential for responsible deployment. The World Health Organization has also provided guidance for the phased testing of genetically modified mosquitoes, emphasizing the importance of thorough risk assessments and community engagement (WHO, 2021). Overall, CRISPR-Cas9 represents an indispensable enabling technology for next-generation insect pest management. Its greatest contribution lies in accelerating functional genomics, validating high-value molecular targets, and providing proof-of-concept for innovative suppression and resistance strategies. Although not currently deployable as a field-ready tool like RNAi, CRISPR continues to shape the conceptual and technological landscape of sustainable, species-specific, and environmentally safer pest control.

**Figure 3:**
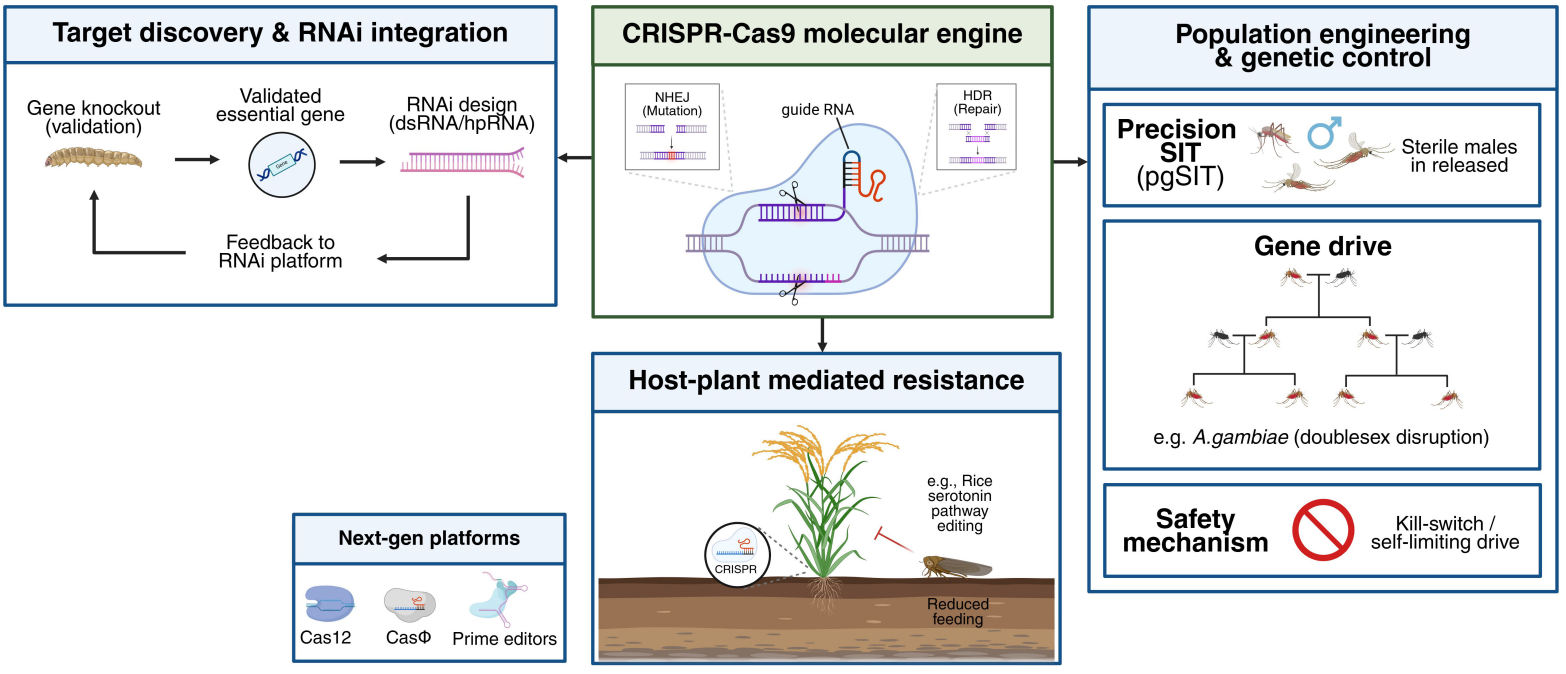
Genome editing in insects.

Under carefully regulated conditions, certain CRISPR-based strategies may evolve into direct control tools. Precision-guided sterile insect techniques (pgSIT) represent a practical extension of the classical Sterile Insect Technique and have been successfully demonstrated in Drosophila species, including the invasive pest *Drosophila suzukii*, with ongoing translational and regulatory efforts toward agricultural deployment (Kandul et al., 2019; Kandul et al., 2022; Yadav et al., 2023). Similarly, CRISPR-based sex ratio distortion systems, such as X-chromosome shredding, and self-limiting or reversible gene drive designs are being developed to enable population suppression while minimizing risks associated with uncontrolled spread (Kyrou et al., 2018; Meccariello et al., 2021; Oberhofer et al., 2020). These approaches are explicitly designed as confined or self-limiting systems, in which genetic effects are restricted to a limited number of generations, preventing long-term environmental persistence. **Figure 3** provides a conceptual overview of genome editing applications in insects, contrasting NHEJ-mediated knockout strategies with HDR-mediated knock-in and gene drive mechanisms. The figure highlights how CRISPR-based tools can be deployed for functional genomics (validating essential genes), population suppression (via targeting sex determination genes such as doublesex), and population modification (e.g., introducing antiparasitic effectors into disease vectors). Together with RNAi, these CRISPR-enabled strategies form a complementary toolkit for sustainable pest management.

In parallel, host plant genome editing offers an indirect but effective avenue for pest management by reducing plant susceptibility or enhancing defense traits without directly targeting insect populations (Faizal et al., 2024).

Importantly, genes validated through CRISPR knockouts can be rapidly prioritized for RNAi-based formulations, ensuring that genome editing and RNAi evolve as complementary and synergistic pillars of smart, next-generation biocontrol.

### 2.5 Sterile insect technique

The SIT is a species-specific and environmentally sustainable pest control strategy that involves mass-rearing, sterilizing, and releasing male insects to suppress wild populations. Released sterile males mate with wild females, resulting in infertile eggs and a gradual decline in pest numbers. SIT has been successfully implemented against several economically significant pests, including the Mediterranean fruit fly (*Ceratitis capitata*), New World screwworm (*C. hominivorax*), and tsetse flies (*Glossina* spp.) (Dyck et al., 2021).

Traditionally, sterilization is achieved using ionizing radiation to induce dominant lethal mutations in the insect germline. However, radiation can compromise male vigor and mating competitiveness, thereby limiting SIT effectiveness. Recent advances in gene editing technologies, particularly CRISPR-Cas9, offer a solution by enabling precise disruption of fertility-related genes while preserving male fitness. For instance, it was demonstrated that targeted disruption of the doublesex gene in *A. gambiae* females resulted in complete sterility, leading to rapid population suppression in cage trials (Kyrou et al., 2018).

Innovations in conditional genetic systems now allow insects to be reared as fertile individuals and rendered sterile only upon release. This approach improves scalability and minimizes the fitness penalties associated with conventional sterilization methods. Further improvements in mass-rearing processes, including optimized diets, automation technologies, and rigorous quality control, help ensure the consistent production of competitive sterile males (Grilli et al., 2021). Release strategies have also evolved, shifting from manual ground-based methods to drone-assisted aerial dispersal, significantly enhancing operational efficiency and coverage (Parker, 2005; Parker et al., 2021). When integrated within area-wide integrated pest management (AW-IPM) programs, alongside biological control agents and targeted pesticide use, SIT contributes to long-term pest suppression while delaying resistance development and minimizing environmental impact (Vreysen et al., 2007).

## 3 Environmental and ethical considerations in genetic pest control

The application of genetically modified (GM) insects and gene drive technologies in pest management introduces complex environmental and ethical challenges. While these biotechnologies present promising alternatives to traditional chemical-based approaches, their potential impacts on ecosystems and societal values require careful evaluation (**Table 3**). A key environmental concern is the unintended effect on non-target species. The release of GM insects may inadvertently impact predators, pollinators, or other organisms within the ecological network. GM insects could affect non-target species via many different routes such as via trophic transfer, through the loss of prey or through indirect behavioral effects-of-natural predators on prey species which may reshape food web structures or change how ecosystems function (Devos et al., 2021). Research and models show that even a highly specialized gene alteration can be transmitted throughout an ecological network for example through cascades of effects occurring via accumulative impacts on trophic layers. The impact of GM insect technology on diversity and stability of communities and the benefit to ecosystem service such as biological control or pollination is multifaceted (Alphey et al., 2020; Courtier-Orgogozo et al., 2017). Transgenic insects, specifically those which have a gene drive mechanism and those which are designed to be self-limiting will yield environment-dependent ecological outcomes that will depend on the environment in which they are placed. These outcomes will be very difficult to predict in field experiments because environmental variation and organism-to-organism interactions is far more complicated than laboratory conditions (Devos et al., 2021). For this reason, recent risk assessment frameworks emphasize that GM insect technologies be assessed ecologically on a case-by-case basis and that the evaluation process includes long-term post-release monitoring of GM insect technologies and an explicit focus on their potential impacts on non-target organisms (Alphey et al., 2020; Courtier-Orgogozo et al., 2017).

**Table 3:** SWOT analysis of genetic engineering strategies in ınsect pest control.

Factor	Strengths	Weaknesses	Opportunities	Threats
Technical	High target specificity; reduced pesticide usage; scalable platforms enabled by synthetic biology	Off-target mutations (CRISPR); inefficient RNAi delivery; resistance development	Integration with AI for real-time monitoring; advanced delivery systems (e.g., nanoparticles, synthetic symbionts)	Horizontal gene transfer; gene drive resistance; regulatory delays
Ecological	Species-specific impact; minimal impact on non-target organisms	Ecological uncertainty of gene drive spread; unclear long-term ecosystem impacts	Precision pest control in complex agroecosystems; suppression of invasive species	Biodiversity disruption; predator-prey imbalance
Economic	Potential for cost-effective large-scale deployment; long-term reduction in pesticide expenditures	High R&D and regulatory approval costs; market access barriers for GM products	Public-private partnerships; biotech investment; increased yield and food security benefits	Disruption to agrochemical markets; monopolization by biotech companies
Societal	Reduced pesticide exposure for farmers; alignment with sustainable agriculture goals	Public distrust in GMOs; misinformation; lack of informed consent in field deployment regions	Inclusive governance models; participatory science; science education and transparency campaigns	Ethical backlash; policy fragmentation; socio-political resistance to genetic interventions
Regulatory	Established frameworks for *Bt* crops and RNAi technologies evolving globally	Lack of harmonized internal regulation for gene drives and synthetic organisms	Opportunity for creating global biosafety protocols; collaborative research programs	Risk of premature release; trade restrictions; delays in crisis response due to legal bottlenecks

### 3.1 Ecological risks and horizontal gene transfer

Horizontal gene transfer (HGT) is a possible ecological risk of GM insects, although this is generally viewed as a low probability risk (Gilbert et al., 2016). HGT of insect–microbe systems can occur via virus mediation (transduction), via mobile genetic elements (plasmids/transposons), or via recombination with symbionts in the gut or gut microbiome (Gilbert and Cordaux, 2013; Soucy et al., 2015). Experimental and genomic analyses show that insects have acquired microbial genes, especially from endosymbiotic bacteria and associated viruses, and this indicates biological feasibility under certain circumstances. However, empirical evidence of the horizontal transfer of engineered transgenes from GM insects to non-targets has yet to be reported in field and semi-field trials (EFSA, 2020). The majority of engineered constructs lack the molecular features needed for independent mobilization (EFSA, 2020) and thus successful HGT would be reliant on a series of low-probability steps such as uptake, genomic integration, and positive selection that would be inferred rather than probed experimentally (EFSA, 2020). Environmental factors such as microbial concentration or viral activity, as well as ecological stress may alter model predictions of the likelihood of this process (EFSA, 2020). A further concern is that of ‘self-propagating’ gene drive elements, spreading beyond their target populations due to this ability. This differs from HGT in risking inadvertent spread by virtue of vertical inheritance but biased transmission; this risk has also been addressed with local or self-limiting drive designs and containment strategies (Noble et al., 2019). Overall the present consensus opinion appears to take this risk seriously but views it as an unlikely risk without specific targeting, falling into the category of being theoretically possible but remote, rather than an empirically documented risk.

*Long-term ecological concerns of gene drives*: Unlike self-limiting strategies such as sterile insect technique (SIT) or precision-guided SIT (pgSIT), CRISPR homing gene drives are designed to propagate indefinitely through wild populations, raising unique long-term ecological concerns (Noble et al., 2019). Once released, a gene drive could potentially spread beyond its target range via migration, wind-assisted dispersal, or accidental transport by human activity, affecting non-target populations of the same species or closely related species (Champer et al., 2020). The evolution of drive-resistant alleles, often generated through non-homologous end joining repair at the target site, could render the drive ineffective or, in some cases, select for undesirable phenotypic traits. Moreover, the potential for irreversible ecosystem alteration such as local or regional extinction of a vector or pest species requires careful consideration of ecological function, food web roles, and the ethical acceptability of intentional species suppression or elimination (National Academies, 2016). Transboundary movement of gene drives raises governance challenges, as no international consensus currently exists on liability, containment, or recall mechanisms. Mitigation strategies, including split drives, daisy-chain drives, reversible drives, and immunizing reversal drives, are under active development but remain largely untested in natural environments (Oberhofer et al., 2020).

### 3.2 Ethical considerations and potential risks

*Informed consent and community engagement:* Informed consent from the community is critical when planning to release genetically modified insects into an ecosystem. Potential long-term effects of the release on the environment, society, and economy will be directly experienced by those within the community. Through open and transparent communication regarding risks and benefits associated with the release of the insects, individuals in the community will be able to be informed participants in the decision-making process and develop a level of trust among themselves and with the researchers, which can lead to a more ethical and socially responsible way of moving forward.

*Animal welfare*: The genetic modification of insects raises questions about their moral and ethical treatment. Although insects are not typically afforded the same ethical considerations as vertebrates, the large-scale manipulation and release of GM insects calls for a reevaluation of their welfare status. *Environmental justice:* Environmental justice concerns arise when the risks and benefits of genetic pest control technologies are unevenly distributed across populations. Marginalized or vulnerable communities, particularly in developing countries where regulatory oversight may be weak, could bear disproportionate burdens if unintended ecological consequences occur such as loss of pollinator services, disruption of local food webs, or transboundary movement of gene drives. Conversely, these same communities may benefit from reduced pesticide exposure and improved crop yields. Ensuring procedural justice (community participation in decision-making), distributive justice (fair allocation of risks and benefits), and recognition justice (respect for local knowledge and values) is essential for ethical deployment (Macer, 2005; Beach and Maselko, 2025).

Transgenic crops have long been the subject of debate among scientists, policymakers, and the public. A major concern is their potential impact on human health, with critics questioning the safety of consuming GM food. Another significant issue is their environmental impact, particularly the potential for transgene flow and its effects on biodiversity.

Séralini et al., reported that rats fed Roundup-tolerant GM maize and/or exposed to Roundup herbicide developed higher rates of mammary tumors, liver congestion, and kidney necrosis (Séralini et al., 2012; Séralini et al., 2014). However, these findings were met with widespread criticism from the scientific community due to the small sample size, lack of statistical power, and the use of a rat strain (Sprague-Dawley) known for high spontaneous tumor incidence. The European Food Safety Authority (EFSA) concluded that the study was inadequately designed and did not provide valid evidence of adverse effects. The broader scientific consensus, supported by hundreds of independent studies, is that currently commercialized GM crops do not pose greater health risks than conventional crops. The broader scientific consensus over the past decade supports the conclusion that diets containing GM food do not pose significant health risks to animals (Kumar et al., 2020; Ortiz and Sansinenea, 2023). Particularly, the food safety of Bt transgenic crops has been extensively evaluated. Key health concerns associated with GM crops include toxicity, allergenicity, and genetic hazards. These are typically assessed through a combination of laboratory studies and controlled field trials before any commercial release.

Approval of transgenic crops requires a rigorous regulatory process that includes environmental risk assessments. Factors such as horizontal gene transfer, pollen viability, and wind dispersal are carefully evaluated. Laboratory and field studies on the environmental and health effects of GM crops have generally shown minimal risk to human health (Meissle et al., 2022; Carrière and Tabashnik, 2023; Tabashnik et al., 2023).

Environmental concerns mainly include gene transfer between crops and wild relatives, as well as effects on non-target organisms. One widely cited example involves *Bt* maize pollen, which was initially believed to harm monarch butterfly larvae (Meissle et al., 2022). To address these concerns, efforts have been made to improve the host specificity and persistence of transgenic traits, aiming to enhance pest control efficacy while minimizing risks to beneficial species and slowing the development of insect resistance (Meissle et al., 2022; Fabrick et al., 2023).

The deployment of genetic pest control technologies raises significant socio-economic and geopolitical questions. Developing countries, where pest burdens are often highest, may lack the regulatory infrastructure, technical capacity, and financial resources to access or evaluate these technologies, creating a dependency on biotech corporations from developed nations. Corporate monopolization of intellectual property (e.g., CRISPR patents, dsRNA production methods) could limit competition, increase costs, and entrench farmer dependency on proprietary seeds and sprays. Disparities in biosafety governance across countries create risks of unregulated deployment, transboundary movement, and environmental injustice. Ethical governance models should include technology transfer agreements, capacity-building initiatives, open-access innovation platforms, and inclusive international frameworks that ensure equitable access and benefit-sharing.

## 4 Conclusions

Over the past two decades, the management of insect pests in agricultural ecosystems has undergone a significant transformation. Molecular genetic tools have introduced an unprecedented level of precision and efficacy, marking a departure from the broad-spectrum and often indiscriminate nature of traditional chemical control. Central to this transformation is the increasing convergence of genetic engineering, functional genomics, and ecological systems thinking.

The deployment of *Bt* crops constitutes a key milestone in reducing dependence on conventional insecticides. These crops have demonstrated reliable efficacy against a wide range of lepidopteran pests, contributing to both increased agricultural productivity and enhanced environmental safety. However, the emergence of resistance, as seen in *H. armigera* and *S. frugiperda*, reinforces a key tenet of pest management: no single intervention remains effective indefinitely. As such, resistance mitigation strategies, particularly toxin pyramiding and structured refuges, must remain central to *Bt* crop implementation.

RNAi-based technologies have introduced a new layer of pest control through gene-specific silencing. This approach offers the potential for highly selective pest suppression with minimal off-target effects. Nevertheless, practical barriers, such as the environmental instability of dsRNA, inconsistent uptake across insect species, and delivery inefficiencies, continue to hinder widespread application. Advances such as nanoparticle-based stabilization and symbiont-mediated delivery systems offer promising solutions to these challenges. Importantly, most sprayable or externally applied RNAi strategies do not involve genetic modification of the crop or the pest, distinguishing them from conventional transgenic (GMO) approaches and potentially easing regulatory and public acceptance hurdles.

CRISPR-Cas9-based genome editing represents one of the most transformative developments in modern pest control. Its capacity to induce inheritable, targeted genetic changes, including knockouts and gene drives, has opened new possibilities for pest population suppression and even eradication. The gene drive targeting the doublesex gene in *A. gambiae* serves as a landmark achievement, demonstrating that vector extinction is technically feasible under controlled conditions. However, due to the ecological uncertainties associated with gene drives, including horizontal gene transfer, unintended gene flow to non-target populations, and the potential emergence of resistance alleles, implementing cautious and phased deployment strategies is crucial.

The SIT, revitalized by CRISPR-based sterilization and aerial delivery systems, is a clear example of how modern genetic tools can enhance established ecological interventions. In parallel, the genetic manipulation of insect symbionts presents an emerging avenue for altering pest fitness and vector competence, with the advantage of being potentially reversible and ecologically contained. Despite their technical sophistication, these tools cannot succeed without public trust, transparent governance, and cohesive regulatory frameworks. Ethical considerations, including biodiversity preservation, community consent in release areas, and the equitable distribution of risks and benefits, must be addressed through proactive engagement. Current regulatory environments are fragmented, and the absence of a global consensus on gene drive deployment poses a significant barrier to coordinated, trans boundary pest management.

Looking ahead, the long-term impact of molecular pest control strategies is likely to diverge based on delivery mode, regulatory acceptance, and resistance dynamics. Sprayable RNAi platforms such as SIGS are expected to follow trajectories similar to selective chemical insecticides, requiring repeated applications but offering high species specificity and flexible resistance management. HIGS-based traits may remain restricted to major crops and high-value systems due to regulatory and public acceptance barriers. CRISPR-based approaches, particularly self-limiting population suppression and modification systems, are likely to remain confined to public health and tightly regulated agricultural contexts, with deployment driven by risk–benefit assessments rather than broad commercial scalability. Products such as Calantha^®^ and Vadescana^®^ represent early indicators that RNAi-based tools may become routine components of integrated pest management, complementing rather than replacing existing biological and cultural control strategies. Over the next two decades, the success of these technologies will depend less on molecular efficacy and more on resistance management frameworks, ecological monitoring, and societal trust.

Looking forward, several emerging trends will shape the next generation of genetic pest control. First, RNAi-based nanobiopesticides represent a critical frontier, where engineered nanoparticles (layered double hydroxides, chitosan, carbon quantum dots) protect dsRNA from environmental degradation and enable targeted delivery. Second, artificial intelligence (AI) is revolutionizing target prediction; machine learning algorithms such as DeepRNAi and AI-guided off-target assessment tools are now capable of predicting optimal gene targets with unprecedented accuracy, reducing experimental trial-and-error. Third, satellite and AI-based pest hotspot mapping allows real-time identification of infestation zones, enabling precision deployment of RNAi sprays or gene drive releases only where needed, thereby minimizing ecological exposure. Fourth, the convergence of nanotechnology, AI, and genetic engineering supports the broader vision of precision agriculture, where pest interventions are data-driven, site-specific, and dynamically adjustable. Finally, the integration of these technologies into sustainable agriculture frameworks will require not only scientific innovation but also adaptive governance, farmer education, and public engagement to ensure equitable and responsible implementation.

In conclusion, while molecular genetic approaches are not a panacea, they represent a powerful and essential component of integrated pest management. Their long-term success will depend not only on scientific and technical progress but also on ecological understanding, inclusive public discourse, and adaptive policy development. As these technologies advance, they must be applied with both scientific integrity and a strong commitment to social and environmental responsibility. While RNAi has already matured into a deployable field strategy, CRISPR’s promise lies in its role as both a transformative enabling tool today and a potential direct control platform of tomorrow.

## 5 Future perspectives

The future of insect pest control will increasingly be in integrated strategies that combine molecular biology, ecological engineering, and precision agriculture. Insects infesting crops rapidly evolve resistance to each single intervention, the Bt crops, and some of the chemical insecticides; single intervention methods have shown their limits and integrated pest management with diversified combinations of interventions will be needed.

RNAi and CRISPR genome editing, base editing, and prime editing technologies that enable more precise engineering. Localized and self-limiting gene drive design address key ecological and ethical issues associated with uncontrolled gene flow. Synthetic biology (engineered microbial symbionts and paratransgenic insects) technologies offer minimally invasive methods of control with high specificity to insects with reversibility. The introduction of smart biocontrol platforms that take into account biosensors, nanotechnology, and data from weather will become an important tool. As pests look for new places to inhabit given climate change once the technologies for pest control have been developed will require predictive modeling and data to support long-term robustness of these technologies. Finally, governance and transparency and ecological risk assessment will need to be paramount throughout the 21st century, and social acceptance matters too, from the standpoint of public acceptance.
